# A Less Virulent COVID-19 Pneumonia

**DOI:** 10.7759/cureus.9426

**Published:** 2020-07-27

**Authors:** Andrew George, Latha Ganti

**Affiliations:** 1 Emergency Medicine, Brown University, Providence, USA; 2 Emergency Medicine, Envision Physician Services, Plantation, USA; 3 Emergency Medicine, University of Central Florida College of Medicine, Orlando, USA; 4 Emergency Medical Services, Polk County Fire Rescue, Bartow, USA

**Keywords:** covid-19, pneumonia

## Abstract

Since its initial outbreak, COVID-19 saw a high death rate with those infected typically presenting with severe respiratory distress along with multi-organ involvement. However, over the course of the pandemic, particularly due to the lower age of those diagnosed with the disease and a greater understanding of the risk posed to certain at-risk populations, a new disease course seems to be more prominent with an overall lower mortality among those diagnosed. We present a typical example of such a case here, showing a less lethal course of COVID-19 occurring in late June amidst the resurgence of new daily cases in the United States.

## Introduction

The novel coronavirus, severe acute respiratory syndrome coronavirus 2 (SARS-CoV-2), outbreak responsible for COVID-19 quickly moved to international significance from the initial cases in Wuhan, reported to the World Health Organization on December 31 [1]. By the end of the next month, 9,976 cases were reported over 21 countries, with the first US case being reported on January 20 [2]. As the situation developed over the next couple months, various measures taken in the United States - including enforced quarantines, cancellations of social events, and other precautions - reduced the number of new cases per day from a high of 43,438 on April 6 to a low of 14,676 on June 3 [3]. State “re-openings” throughout mid-to-late June into July appear to have triggered a new resurgence in new cases, with around 55,000 new cases daily in early July [4].

However, data suggest that for a variety of reasons, the course of typical COVID-19 outcomes in the most recent month differs from when the virus first came to the country. With the average age of diagnosed patients decreasing, the ratio of deaths to cases appears to be dropping, with even recent hospital visits resulting in discharge after a brief stay in the emergency department (ED) [5].

We present one such case of a late middle-age male presenting to the ED with a case of COVID-19 pneumonia but being well enough to go home from the ED. Such cases are becoming more common in this resurgence of COVID-19 and may very well represent the new expected course of the disease. Moreover, the use of CT in this case is typical of a practical means of diagnosing COVID-19 in emergency settings, where traditional real-time polymerase chain reaction (RT-PCR) tests may be unavailable [6].

## Case presentation

In late June, a 51-year-old male presented to the ED with a chief complaint of shortness of breath. The patient reported sporadic fevers and chills at home over the past two weeks, but denied current fever, nausea, vomiting, diarrhea, abdominal pain, headache, or any urinary symptoms. The patient reported no past medical history and was not on any medications. His son, however, was diagnosed with COVID-19 two weeks prior, with a cough, fever, and typical viral syndrome type symptoms. The patient reported that he did not quarantine from his son even after the positive COVID-19 diagnosis, as they lived in the same house.

**Figure 1:**
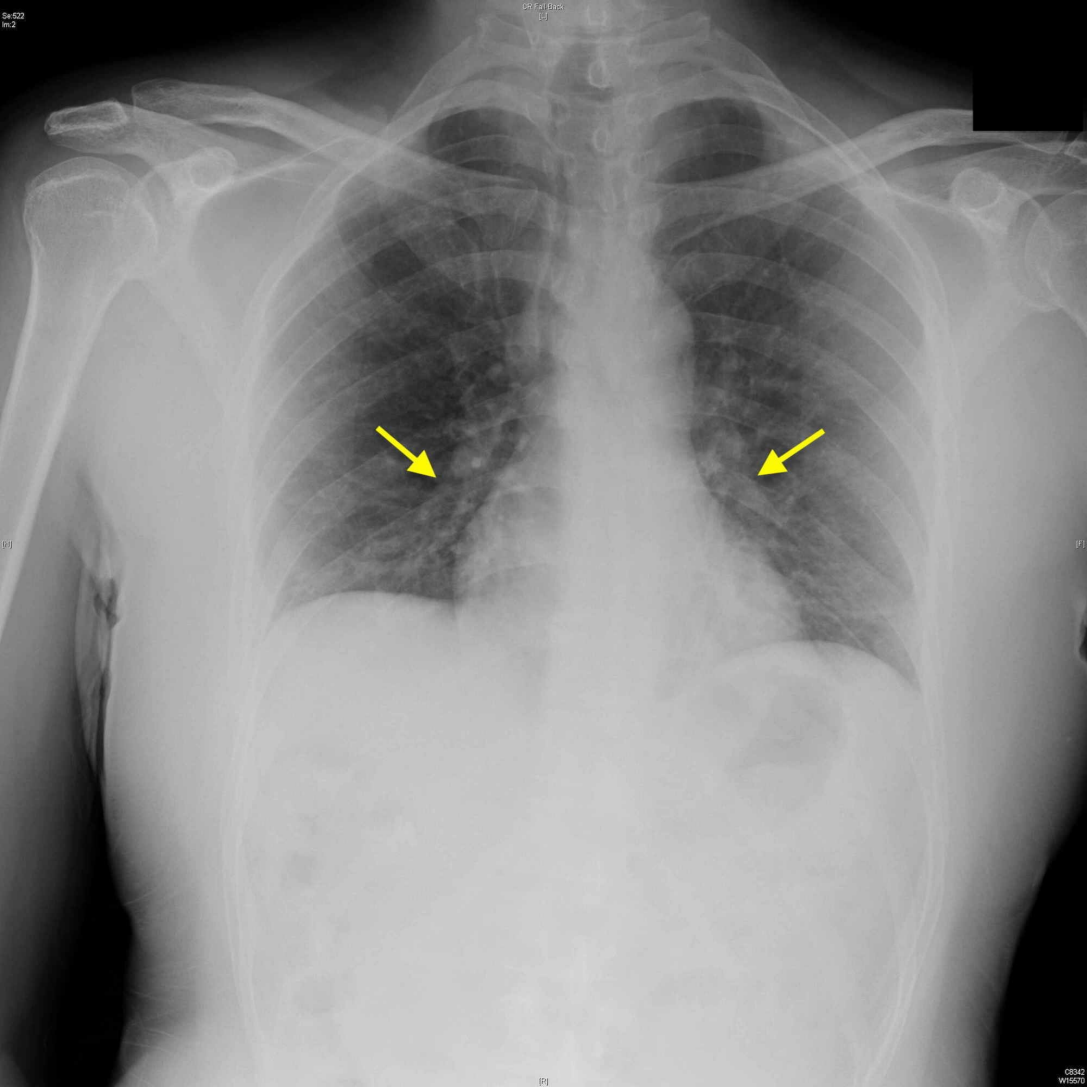
Chest radiograph demonstrating bilateral opacities associated with multifocal pneumonia (arrows)

The patient’s vital signs were temperature 99.1°F, blood pressure 111/76 mmHg, pulse rate 87 beats per minute, respiration rate 18 breaths per minute, and oxygen saturation of 94% on room air. Physical examination revealed significantly decreased breath sounds bilaterally. The remainder of physical examination was unremarkable, including cardiovascular and neurological examination. Laboratory analysis showed signs of leukopenia (white blood cell count of 3,000 per cubic mm) but was negative for neutrophilia or lymphocytopenia. All electrolytes were within normal limits. The D-dimer count was elevated at 2.05 mg/L. Radiological examination included a chest x-ray that demonstrated scattered opacities throughout both lungs suggestive of multifocal atypical pneumonia (Figure 1).

**Figure 2:**
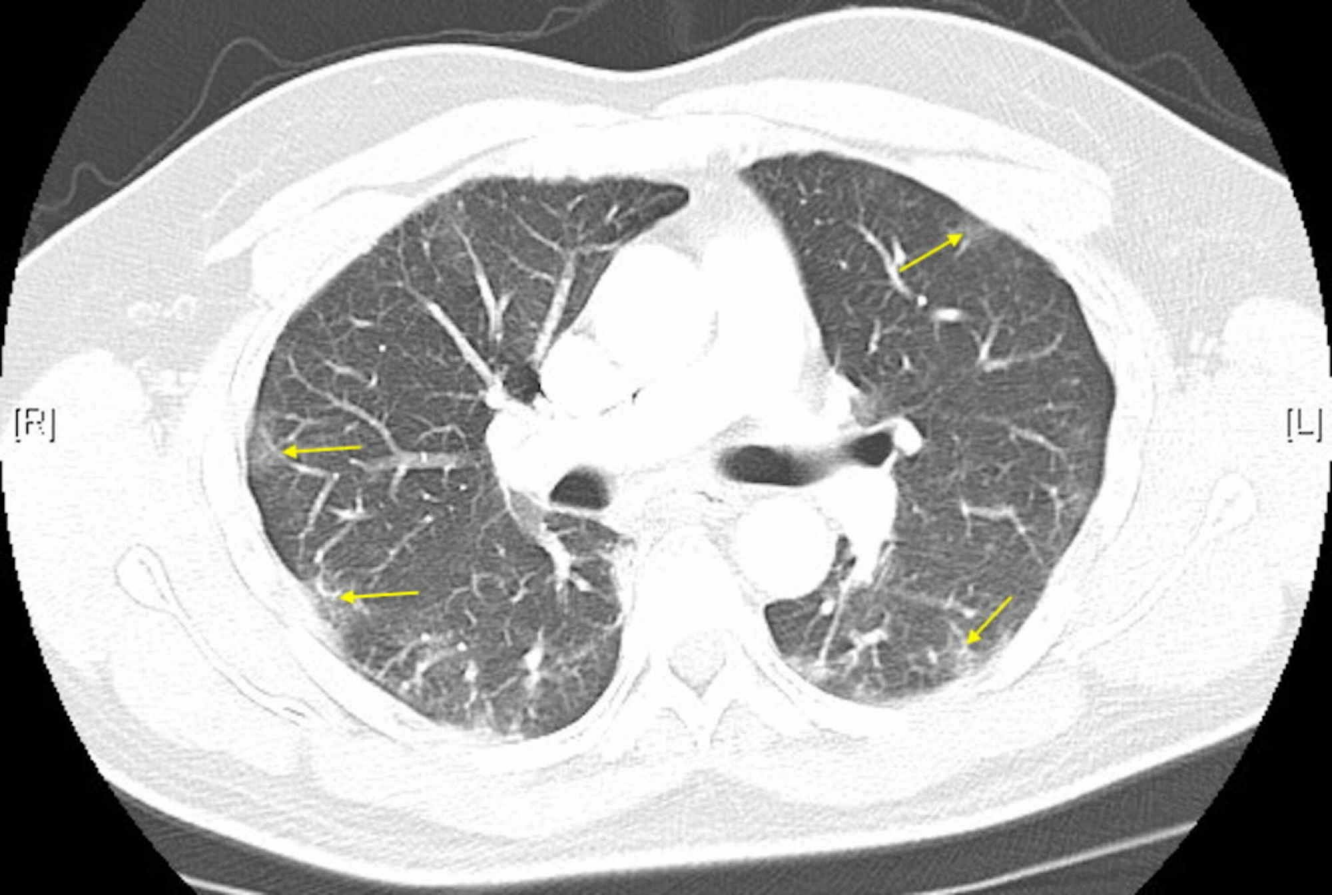
Chest CT demonstrating bilateral ground glass opacities

Chest CT showed multifocal ground glass opacities (GGO) throughout both lungs typical of underlying SARS-CoV-2 infection. There was no evidence for pulmonary embolism (Figure 2).

He appeared well throughout the remainder of his stay in the ED with stable vitals. The patient was ambulated in the ED with no decrease in oxygen saturation noted. Although the patient had imaging findings typical of COVID-19 pneumonia and a corroborating history of present illness, the patient was nonetheless treated with 500 mg intravenous azithromycin and 1 g of ceftriaxone as empiric treatment for community-acquired pneumonia. Given that the patient had no other comorbidities, and wanted to go home, he was discharged home with a course of oral antibiotics. During a telephone follow-up three days after being discharged, the patient reported that he continued to feel better while at home.

## Discussion

CT scans have recently been embraced by EDs where RT-PCR and sequencing are not available, possibility due to a lack of availability [5]. Moreover, its high sensitivity in diagnosing COVID-19, 97% in a study of 1,014 patients, makes it a useful diagnostic tool even when RT-PCR is available, as the same study reported a five-day interval between initial negative and initial positive RT-PCR tests [6].

Although there exists some variability in CT scan findings across patient cases, GGO remains the main CT feature, with involvement in multiple lobes in most cases [7]. Bilateral involvement and peripheral distribution are typically observed, and consolidation is observed in some cases as well [5].

COVID-19 presentation typically also includes some form of respiratory distress, with common clinical features across the pandemic including fever, coughs and sore throat, headaches, myalgia, and shortness of breath [8]. Myalgia, dyspnea, expectoration, diarrhea, nausea and vomiting, and general gastrointestinal involvement have also been reported as common clinical symptoms in patients [9]. Laboratory results often show leukopenia and elevated D-dimers [10].

**Figure 3:**
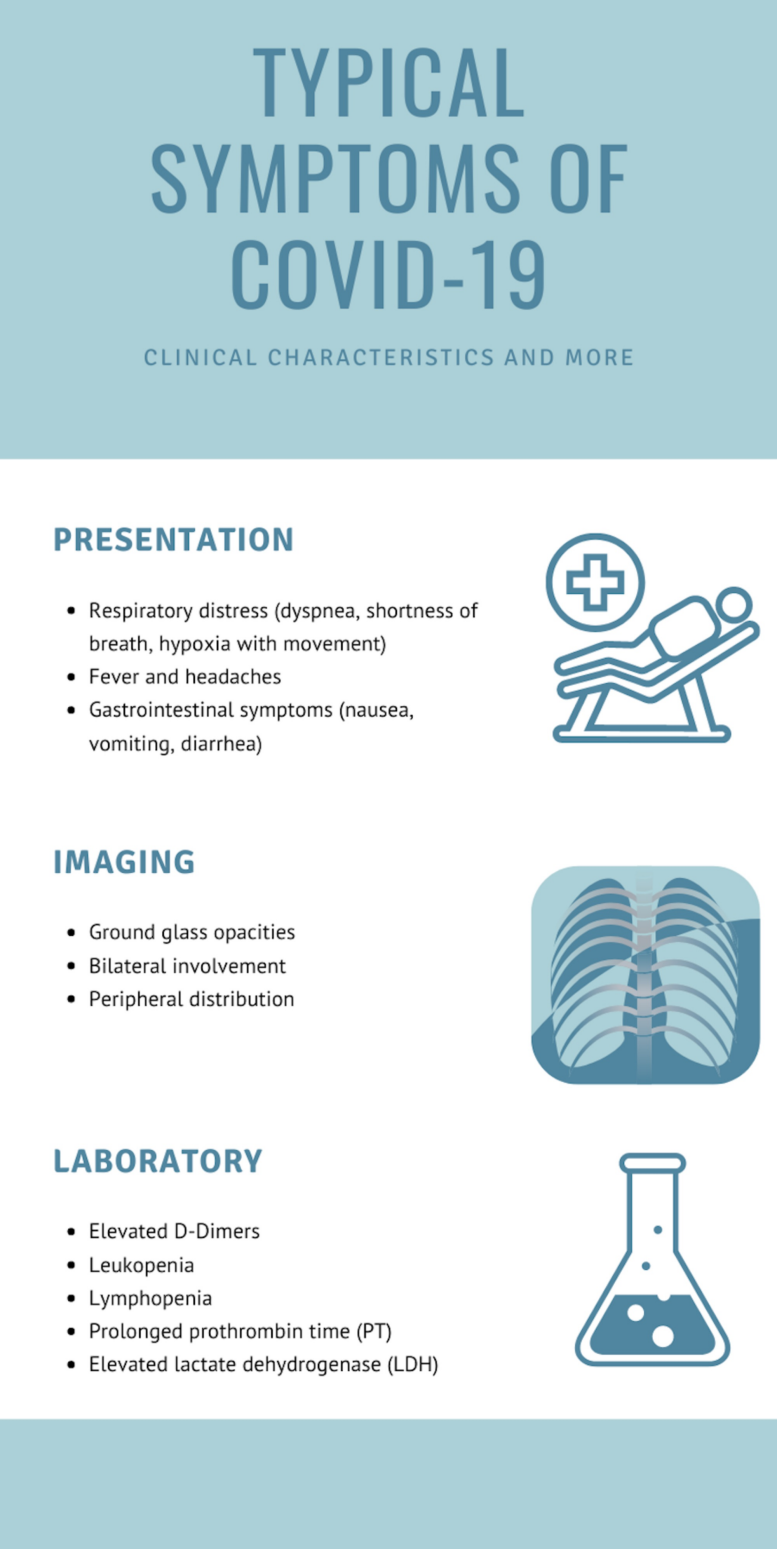
Common findings associated with COVID-19 pneumonia

The case presented shows a patient notable for some of the common COVID-19 symptoms, showing on-and-off fevers and chills, shortness of breath, elevated D-dimers, and leukopenia, but no additional organ system involvement (including gastrointestinal), no dyspnea or acute respiratory distress, no myalgia, and a lack of other common symptoms (Figure 3).

The patients x-ray and CT scans reveal multifocal GGO. Nonetheless, the patent was able to go home from the ED. On three-day telephone follow-up, the patient was still managing okay at home. 

Although this case shows typical attributes of COVID-19 viral pneumonia, the overall severity of this case is lower than literature tends to suggest to date. While many factors are at play in the lower ratio of COVID-19 related deaths to cases, it is entirely possible that as new cases are encountered in less at-risk populations, the course of the disease will be more akin to what we have observed. Although the one case observed here provides only a single point of anecdotal evidence, taken in combination with the lowering ratio of deaths to cases it may suggest the importance of observing the characteristics and symptoms of COVID-19 over the course of the pandemic and determining whether, since the initial outbreak and arrival in the country, a significant change in the lethality of the disease exists and for what reasons.

This more benign form of pneumonia may reflect a lower virulence of the current COVID-19 strain, or may reflect patient factors. Some reported risk factors through to contribute to COVID-19 morbidity include obesity, smoking, alcoholism, and poor overall health. Our patient had a normal BMI, did not smoke cigarettes or drink alcohol, and had no medical history. Thus, despite his age of 51 years, he was overall healthy and presumably able to combat the virus well.

## Conclusions

This case reports on a seemingly less virulent COVID-19 pneumonia, with a more benign course compared to the COVID-19 pneumonias we saw several months ago. This may be due to characteristic associated with the virus itself including mutation, or may have to do with patient factors. It is important to recognize various patterns in this evolving pandemic. 
